# Major depression revealing primary hyperparathyroidism: A case report

**DOI:** 10.1192/j.eurpsy.2021.902

**Published:** 2021-08-13

**Authors:** R. Ouali, M. Turki, S. Ellouze, T. Babah, R. Charf, N. Halouani, J. Aloulou

**Affiliations:** 1 Psychiatry B Chu Hedi Chaker, Tunisia, psychiatry B, sfax, Tunisia; 2 Psychiatry (b), Hedi Chaker University hospital, sfax, Tunisia; 3 Psychiatry (b), Psychiatry (B), Hedi Chaker University hospital, sfax, Tunisia

**Keywords:** hyperparathyroidism, Depression

## Abstract

**Introduction:**

Psychiatric symptoms associated with Primary hyperparathyroidism (PHPT) involved several presentations; the most characteristic is depression. However, PHPT remains often overlooked by physicians when making differential diagnosis for patients with psychiatric disorders, particularly in the elderly.

**Objectives:**

We proposed to describe the clinical and therapeutic characteristics of major depression secondary to PHPT.

**Methods:**

We report a case of PHPT revealed by depression. Then, we conducted a literature review using “PubMed” database and keywords “primary Hyperparathyroidism”, “depression”.

**Results:**

A 73-year-old man presented with a 3-month history of depressed mood, loss of interest, clinophilia, poor concentration, and weight loss. These symptoms were associated with epigastralgia and constipation not responding to symptomatic treatment. The etiological assessment was normal. The diagnosis of major depression was established, and the patient was treated with Sertraline (25 mg/day). After one month of treatment, somatic and psychiatric symptoms worsened. Physical examination revealed a deteriorated general condition, dehydration, and cardiac arrhythmia. Blood analysis revealed renal failure, hypercalcemia (4.2mmol/L), hypophosphatemia (0.4mmol/L), and increased parathyroid hormone level (180 pg/ml). The patient was hospitalized in intensive care unit. Cervical echography showed 2 hyperparathyroid adenomas, and diagnosis of PHPT was established. Under symptomatic treatment, the patient’s somatic and psychiatric condition improved. An hyperparathyroidectomy is undergone soon.

**Conclusions:**

This case highlighted the importance of considering a primary psychiatric disorder as a diagnosis of exclusion, especially in the elderly. PHPT is one of differential diagnoses for psychiatric symptoms, like depression, whose management is conditioned by that of the somatic disease.

